# Exploring the Objective Signs of Imminent Suicide Risk in Psychiatric In-patients

**DOI:** 10.14789/jmj.JMJ22-0002-OA

**Published:** 2022-06-09

**Authors:** HIROMI SHIMAZAKI, ITARU UTAGAWA, CHIEMI SANO, SHINOBU SAKURADA, KYOUHEI YAMASHITA, NOBUTO SHIBATA, TUNEYOSHI OTA, TADASHI FUKUSHIMA

**Affiliations:** 1 Graduate School of Health and Sports Science, Juntendo University, Chiba, Japan; 1 Graduate School of Health and Sports Science, Juntendo University, Chiba, Japan; 2 Asahinooka Hospital, Kanagawa, Japan; 2 Asahinooka Hospital, Kanagawa, Japan; 3 Home-Visit Nursing Station, Asuka, Tokyo, Japan; 3 Home-Visit Nursing Station, Asuka, Tokyo, Japan; 4 Department of Psychiatry, Juntendo Tokyo Koto Geriatric Medical Center, Tokyo, Japan; 4 Department of Psychiatry, Juntendo Tokyo Koto Geriatric Medical Center, Tokyo, Japan; 5 Department of Psychiatry, Juntendo University School of Medicine, Tokyo, Japan; 5 Department of Psychiatry, Juntendo University School of Medicine, Tokyo, Japan

**Keywords:** suicide, in-patient, psychiatric hospital, objective signs

## Abstract

**Objective:**

This study retrospectively explores the objective signs of imminent suicide risk in psychiatric in-patients.

**Design:**

The study analysed the diagnostic and nursing records of a psychiatric hospital that covered the last 14 days before the suicide attempts of 18 people, who, between March 2008 and July 2019, were found to have died by suicide during their hospital stay.

**Methods:**

Three professionals used a fishbone diagram to separately identify the factors that led to each person’s suicide, the objective signs that indicated imminent suicide risk, possible preventive strategies, and other observations. They compared their findings and used the KJ method (Kawakita Jiro Method) to categorise the items on which they all agreed.

**Results:**

Objective signs of imminent suicide risk were condensed into five categories: ‘signs emanating from the patient’, ‘signs gleaned through engagement’, ‘signs from response to treatment’, ‘signs associated with reports from the family’, and ‘signs inferred from multiple sources of information’. Five categories describing issues with the way in which the hospital staff handled information were extracted, namely ‘omission in diagnostic records during admission’, ‘omission in conference records’, ‘communication lapse during transfer’, ‘need for integrated information’, and ‘systemic issues’.

**Conclusions:**

The findings offer insights on assessing suicide risk and preventing suicide.

## Introduction

Psychiatric hospitals play an important role in suicide prevention by protecting and treating people who present a risk of suicide. However, suicides still occur among psychiatric in-patients, with an estimated suicide rate of 147 per 100,000 patients^1)^. Aside from preventing any possibility of treatment, suicides inflict severe trauma on the patients’ families and the people around them, and potentially expose the hospital to litigation. It is therefore essential to prevent suicides among psychiatric in-patients. Although factors causing suicide have been investigated for a long time, the results derived so far present limited sensitivity and low positive predictive value of the combinations of risk factors, suggesting that suicide risk models have limited use in clinical settings^2-4)^. For example, Neuner et al. (2008) identified four risk factors of suicide among in-patients, including resistance to psychopharmacological treatment and past suicide attempts, but these could explain only 10% of the variance^5)^, and a study by Powell et al. (2000) similarly found that predictors correctly identified a mere 2% of suicide patients^6)^. Chronic risk factors such as past suicide attempts and mental illness have been considered ill-suited by practitioners from the perspective of clinical effectiveness, as such risk factors cannot assess suicide risks that fluctuate over time. Recently, however, studies that have highlighted the value of proximal, near-term risk factors have been gaining attention. For example, Rudd et al. (2006) proposed a set of warning signs of suicide risk in the near term─in time frames of minutes, hours, and days^7)^. Yaseen et al. (2019) proposed diagnostic criteria for predicting imminent suicide risk, namely the suicide crisis syndrome (SCS)^8)^. However, one issue is that the warning signs do not necessarily arise only immediately before death by suicide^9)^. One issue with the SCS is that it predicts post-discharge suicidal behaviour only in a limited number of cases^8)^.

Other authors have suggested that as interventions based on clinical risk assessments are insufficient in preventing suicides among psychiatric in-patients, it is necessary to focus on a mix of other strategies, such as providing a safe environment and observing patients effectively^10-12)^. Menon (2013) argued that suicide risk should be assessed in three areas, namely risk factors, warning signs (current mental state), and protective factors^4)^. However, he did not clarify the warning signs that are particular to psychiatric in-patients. Clinical inquiries, structured interviews, and self-assessment questionnaires have limited value in assessing suicide risk, given that some patients have attempted suicide despite denying having suicidal ideation^13, 14)^ and in cases where the patient has trouble communicating or shows a decline in comprehension, or has limited trust in healthcare professionals. It is possible to obtain information through alternative means, such as by having ward staff monitor the in-patients’ behaviour or chat with them. Thus, it should be possible to improve the precision of risk assessments by looking out for specific objective signs that indicate imminent suicide risk in the in-patients’ behaviour in the ward, instead of relying on diagnostic records alone. We conducted a retrospective study of the objective signs of imminent suicide risk among psychiatric in-patients as drawn from a combination of diagnostic and nursing records (nurses’ recorded observations of patient behaviour in the ward).

## Materials and Methods

### Sample

We analysed the diagnostic and nursing records of 18 in-patients (6 male, 12 female) maintained by a psychiatric hospital. These people were found to have died by suicide during their hospital stay, between March 2008 and July 2019 (11 years and 4 months). In each case, the surviving family members did not object to their data being used in this study. The sample included cases in which the patient died by suicide during temporary leave and in which the patient was rushed to hospital after a suicide attempt and subsequently died in the hospital. It may be unclear in some cases as to whether the death really was by suicide or accident (the person is no longer around to confirm this). Accordingly, in this study, we defined suicide as an event in which the person committed an act that resulted in their death, irrespective of whether or not they anticipated this fatal outcome^15)^. We considered utterances, behaviours, attitudes, and mental states observed over a period of 14 days before the patient's fatal suicide attempt as signs of imminent suicide risks.

### Preparing the Data to be Analysed

In Resource A, we compiled the patients’ basic information, which comprised diagnostic records made at time of admission, their diagnosis, period of hospitalisation, and history of suicide attempts, psychiatric symptoms, suicide in their families, and diseases. In Resource B, we arranged the diagnostic and nursing records into a time series for a period of 14 days before the fatal suicide attempt. In both Resources A and B, all data were anonymised.

### Analysis 1

From Resources A and B, we aggregated the cases in which the patient had attempted suicide in the past; the patient’s problem list at the time of admission mentioned suicidal behaviour or ideation; and the patient showed signs of suicide in the 14 days before the fatal suicide attempt, including suicidal utterances, self-injury, and unsuccessful suicide attempts.

### Analysis 2

Analysis 2 was conducted by three professionals from the field of psychiatric care (‘professionals’), namely a doctor, a nurse, and a clinical psychologist, each of whom had at least 10 years of experience in clinical psychiatric care, including ward duty. To ensure objectivity, none of the professionals had engaged in any way with the cases in our sample. The three professionals were provided with Resources A and B in advance and were asked to analyse three sets of items using a fishbone diagram (a diagram for analysing the potential causes of an outcome, which is often used to analyse the causes of accidents). The three sets of items were: factors that led to each person’s suicide (C), imminent suicide signs (D), and preventive strategies or other observations (E). The professionals conducted this analysis separately and then came together to compare and consolidate their findings for C, D, and E. The items they all agreed upon (F) were set aside for further analysis.

### Analysis 3

Analysis 3 focused on F, as derived through analysis 2. The imminent signs and risk factors were labelled/encoded and organised by content using the KJ Method (where data are sorted into groups that present relationships of affinity, opposition, and causality in order to generate a hypothesis). We prioritized reducing omission of behaviours that could be signs, and as such included two behaviours that the three professionals had not agreed upon but had all noted, and one sign that, although not noted by the professionals, was evidently a sign of imminent suicide risk.

### Analysis 4

We considered F in terms of the human factor, such as cases where the staff failed to record important patient information. We labelled/encoded items describing the handling of patient information by the hospital staff and categorised them using the KJ Method.

### Ethical Considerations

This study was approved by the psychiatric hospital in question (approval number: 2) and the ethics committee of the Graduate School of Health and Sports Science at Juntendo University (approval number: 31-74).

## Results

### Patient Characteristics

Table 1 shows the patients' characteristics based on Resource A: sex, age, the diagnosis stated in the medical records, the retrospective diagnosis given by the three professionals in Analysis 2, and the period of hospitalisation.

**Table 1:** Patients' characteristics

Id	Sex^a)^	Age	Diagnosis in medical records	Diagnosis by analysis 2	Period of hospitalisation (day)
1	F	21	Depressive Disorder	Possibility of Personality Disorder	2
2	M	21	Schizophrenia		20
3	F	27	Borderline Personality Disorder		40
4	M	33	Adjustment Disorder, Narcissistic Personality Disorder		104
5	F	34	Dissociative Disorder	(Complex-PTSD or other trauma-related conditions)	30
6	F	40	PTSD, Bipolar II Disorder	Not PTSD, but Acute Stress Disorder?	1
7	F	43	Schizophrenia		916
8	F	48	Schizophrenia		2416
9	M	53	Schizoaffective Disorder		99
10	F	54	Bipolar II Disorder		17
11	F	54	Depressive Disorder		40
12	F	55	Depressive Disorder		61
13	M	56	Bipolar I Disorder		17
14	M	63	Recurrent Depressive Disorder		3
15	M	67	Depressive Disorder (suspected of having Dementia with Lewy bodies)		147
16	F	69	Depressive Disorder (suspected of having Front-temporal or another form of Dementia during the intake interview)	Possibility of Bipolar Disorder (The principal doctor described ‘elevated mood’ and ‘unusual talkativeness’ among other things)	3
17	F	69	Depressive Disorder		79
18	F	79	Schizophrenia	Not Schizophrenia, but Depressive Disorder? (The principal doctor had prescribed medication for depression)	7001

a) M: Male, F: Female

### Rates of suicidal ideation, self-injury, and suicide attempt (Analysis 1)

Of the 18 cases analysed, 11 (61.1%) had a problem-list entry of attempted suicide or suicidal ideation at the time of admission, and 11 (61.1%) had a past suicide attempt more than two weeks before the suicide attempt that led to death (or before hospitalisation if the hospitalisation period was less than fourteen days). Further, in the two weeks preceding their death (or during the hospitalisation period if the hospitalisation period was less than fourteen days), 7 (38.9%) had made suicidal utterances, 2 (11.1%) had self-harmed, and 3 (16.7%) had attempted suicide unsuccessfully, while 11 (61.1%) had had no suicidal utterances or suicide-related behavior.

### Objective Signs of Imminent Suicide Risk (Analysis 3)

Table 2 shows the major categories, sub-categories, and sub-sub-categories of the signs indicating imminent suicide risk, along with examples. In the following, the major categories are denoted by ‘ ’, sub-categories by [ ], sub-sub-categories by < >. Our analysis yielded five major categories. The first one, namely ‘signs emanating from the patient’, describes signs that the patient expresses spontaneously, without any engagement from the hospital staff. The second, namely ‘signs gleaned through engagement’, describes signs that the hospital staff glean through their engagement with the patient. The third, namely ‘signs from response to treatment’, describes signs that the patient exhibits in response to medical intervention. The fourth, that is, ‘signs associated with reports from the family’, describes signs reported by the patient's family. The fifth, namely ‘signs inferred from multiple sources of information’, describes signs that are inferred from an integrated analysis of diagnostic and nursing records along with other information on the patient.

**Table 2:** Objective signs of imminent suicide risk (Analysis 3)

Major category	Sub-category	Sub-sub-category	Example
Signs emanating from the patient	Behaviour	Suicide attempt	Twelve days before the event, the patient attempted suicide by swallowing rat poison.
		Self-injury	On the day before the event, the patient hurt their right wrist with their fingernail.
		Morbid behaviour	The patient repeatedly recited Buddhist sutras beginning five days before their suicide.
		Accesses means of suicide	The patient tried to open a window in the closed ward.
		Desire to leave	On the day of the event, the patient tried to escape from the closed ward.
			During the patient’s last several days, they repeatedly applied for temporary leave, only to cancel it.
		Reticence	The patient discussed their suicidal ideation in the past, but never mentioned a desire to die in their last four days.
	Symptoms	Sudden deterioration	The patient’s schizophrenic symptoms suddenly deteriorated.
		Symptoms aggravated by discharge anxiety	The patient had no social relationships. Ten days prior to their death, the patient discussed their anxiety about the upcoming discharge. Subsequently, the patient experienced an escalation in anxiety, dread, and dissociation.
		Unstable symptoms	The patient experienced intra-day fluctuations in depressive disorder (cycling between gleeful and gloomy utterances).
Signs gleaned through engagement	Verbal information	Talks about suicidal ideation	The patient repeatedly told a nurse that they wanted to die because they could not bear the hallucinations and other psychiatric symptoms, and because of the self-disgust they felt from when the symptoms worsened.
		Talks about suicidal ideation due to proactive communication	The patient booked a period of leave, but on the day before the event, they expressed second thoughts. Noticing that the patient had not left their room since then, the nurse entered the room. The patient remained silent and pensive for 15 minutes, before declaring that they had something to say. The patient then admitted that they had reserved the period of leave out of a desire to die.
		Declares suicidal intention	On the day of the event, the patient, without returning to the ward, made a silent phone call to the hospital. The staff returned the call and the patient answered. When asked for their location, the patient said that they did not want to give it and that they no longer needed any food as they were about to die. ^a^^)^
		Complains of loneliness	The patient complained of loneliness and a sense of not fitting in anywhere. The patient also mentioned poor marital relationships.
		Communication difficulties	The patient experienced a sudden deterioration in schizophrenic symptoms. An entry in the nursing record made on the day before the event reads thus: ‘The patient is making no sense’. It also means that the patient’s condition had become worse.
	Nonverbal information	Reluctant expressions	On the day of the event, the patient was taken to a seclusion room and told that her bra would need to be removed. On hearing this, the patient gave a grudging grimace.
		Evasiveness	The patient tried to hide when they were called by the principal doctor, and drew back during temperature tests. ^b^^)^
		Poor treatment motivation	The patient had no desire to get better.
		Resists treatment	Lacking awareness of the fact that they were ill, the patient denied experiencing psychiatric symptoms or suicidal ideation and repeatedly refused treatment, while demanding to be discharged.
		Fails to understand the importance of treatment	The patient failed to recognise that they were admitted because they had attempted suicide, and that they had failed to comprehend the importance of treatment.
Signs from response to treatment	Response to learning of diagnosis		The patient was informed of their diagnosis four days before the event. The patient struggled to come to terms with the diagnosis, saying that they were in a state of shock and wanted to find a way to ease it.
	Response to clinical environment	Response to admission	The patient had never been admitted to a psychiatric hospital before and experienced anxiety and dread in the new environment.
		Response to ward transfer	Deemed a suicide risk, the patient was transferred to a closed ward. Although this was necessary, the new environment caused the patient’s psychotic symptoms to worsen.
	Response to dosage change		The patient’s dose was decreased because of side-effects. Consequently, the patient experienced anxiety and irritability, symptoms that were suppressed by the medicine or withdrawal symptoms.
	Unnatural clinical response		Following the first session of ECT after resuming therapy, the patient exhibited lively behaviour that was incongruous with their behaviour theretofore. For example, after drinking something, the patient gleefully declared that the drink was delicious. They also purchased a sports drink and consumed 90% of a curry. ^b^^)^
Signs associated with reports from the family	Reports from the family	Report on suicidal ideation	The family informed the staff that the patient had thought about suicide.
		Information provided during the meeting	On the day of the event, the family informed the staff that the patient was crying after their meeting. A nurse visited the patient’s room, and the patient lamented that their hopes of discharge had been dashed.
	Family report not discussed		The family informed the staff about the patient’s suicidal ideation. However, the staff neglected to discuss this with the patient.
Signs inferred from multiple sources of information	Attitude inferred from integrated information	Tries to please the principal doctor	What the patient talked about and how they behaved varied between the principal doctor and nurses. In the presence of the principal doctor, the patient tried to give the impression that they were getting better. In contrast, with the nurses, the patient complained of anxiety and anxiety-induced somatic conditions.
	Situational inferences	Isolation	The family said that they would not allow the patient to return to the family home after discharge.

a) Behaviour not noted by the three professionals during Analysis 2 (we subsequently added them) b) Behaviour that was focused on during Analysis 2 but for which the three professionals’ interpretations were different

The first major category contained two sub-categories, namely [behaviour] and [symptoms]. [Behaviour] contained six sub-sub-categories, namely <suicide attempt>, <self-injury>, <morbid behaviour> (such as reciting sutras), <accesses means of suicide>, <desire to leave> (escape attempts, hesitating to stay overnight), and <reticence> (among patients who had been complaining of suicidal ideation). The second sub-category, [symptoms], contained three sub-sub-categories, namely <sudden deterioration> (in schizophrenia), <symptoms aggravated by discharge anxiety>, and <unstable symptoms> (intra-day fluctuations in depressive disorder).

The second major category contained two sub-categories, namely [verbal information] and [nonverbal information]. [Verbal information] contained five sub-sub-categories. The first three described explicit verbal communication from the patient: <talks about suicidal ideation>, <talks about suicidal ideation due to proactive communication>, and <declares suicidal intention>. The fourth sub-sub-category is <complains of loneliness>. The final one is <communication difficulties>, which implies a possible deterioration in symptoms. The other sub-category, [nonverbal information], contained five sub-sub-categories. Four of these described behaviours and attitudes, namely <evasiveness>, <poor treatment motivation>, <resists treatment>, and <fails to understand the importance of treatment>. The fifth sub-sub-category, <reluctant expressions>, described the patient’s feelings of reluctance toward safety restrictions on objects that patients could use to kill themselves.

The third major category, ‘signs from response to treatment’, contained four sub-categories. [Response to learning of diagnosis] described cases in which the patient had just learned of their diagnosis and struggled to come to terms with it; [response to clinical environment] described responses to being admitted to hospital or being transferred to a closed ward; [response to dosage change] described symptoms associated with a reduction in the dosage following an adverse reaction. The fourth sub-category, [unnatural clinical response], described a case in which a patient who had discontinued electroconvulsive therapy (ECT) exhibited, upon resuming ECT, animated behaviour that was incongruous with their behaviour theretofore.

The fourth category, ‘signs associated with reports from the family’, contained two sub-categories. The first, [reports from the family], included <report on suicidal ideation> and <information provided during the meeting (with the patient’s family)>. The second, [family report not discussed], described cases in which, despite being informed by the family of the patient’s suicidal ideation, the staff neglected to discuss it with the patient.

The fifth category, ‘signs inferred from multiple sources of information’, contained two sub-categories: [attitude inferred from integrated information] and [situational inferences]. The former contained the single sub-sub-category <tries to please the principal doctor>, which described cases in which the attitude only became apparent once the staff had integrated diagnostic and nursing records, and the patient behaved as though he/she was getting better only in the presence of the doctor, in order to meet the doctor’s expectations. The latter contained the single sub-sub-category <isolation>, which described cases in which the patient was assumed to be isolated given that the family refused to let the patient live with them after being discharged.

### Issues in the Manner in which the Hospital Staff Handle Information (Analysis 4)

Table 3 presents the major categories and sub- categories drawn from Analysis 4, along with examples. Our analysis yielded five major categories: ‘omission in diagnostic records during admission’, ‘omission in conference records’, ‘communication lapse during transfer’, ‘need for integrated information’, and ‘systemic issues’.

**Table 3:** Issues in the way in which the hospital staff handle information (Analysis 4)

Major category	Sub-category	Example
Omission in diagnostic records during admission	Omission in diagnostic records and treatment policy	The diagnostic record made for admission omitted the diagnosis and treatment policy.
	Omission in patient information during readmission	The patient was readmitted just four days after being discharged. The diagnosis record for readmission omitted any opinion on the recurrent symptoms and suicidal ideation. It also omitted the treatment plan. The diagnostic record for the original admission was in a separate document.
	Abridging report on suicide risk	In the record for admission, the doctor mentioned suicidal ideation despite the patient denying it. However, the record contained no opinion on the occurrence of suicidal ideation.
	↓	
	Factors influencing ward staff’s judgements and behaviours	While delivering the medication, the nurse noticed that the patient was missing. The nurse knocked on the lavatory door and there was no response. The nurse finished delivering medication to the other patients and then looked for the missing patient, whom they discovered hanging in the lavatory. The nurse may have elected to leave the area (instead of searching for the patient immediately) because the severity of the suicide risk was not fully appreciated.
Omission in conference records	Omission in treatment policy	The conference records omitted the treatment plan.
Communication lapse during transfer	Hard to relay complex information such as the principal doctor’s decision-making process	The patient had been transferred from a seclusion room in an open ward to the general area of the closed ward for their protection following a suicide attempt. While bathing alone, the patient attempted suicide by swallowing shampoo and conditioner. The staff in the destination ward may have assumed that the patient no longer needed to be secluded because their psychiatric condition had improved, and they may have got this impression because the staff failed to share the intent of the principal doctor’s orders.
	Omission in transfer application records	Nine days before the event, the patient tried to hang themselves. Seven days before the event, the patient, in a seclusion room, banged their head against the wall and toilet out of a desire to kill themselves. One day before the event, the patient was transferred. The principal doctor’s transfer application did not mention the suicide attempts.
Need for integrated information		Despite the nursing records having plenty of information on patient complaints and symptoms, the patient, during medical screenings, was judged as stable. Accordingly, the form of hospitalisation was changed and the patient was given more freedom. Decisions should be based on the information in both the nursing and diagnostic records.
Systemic issues	Hard to view records	It is hard to share the records among the staff, as they comprised a single paper document.
	Hard to access past information	The patient died by suicide by swallowing detergent after their delusions worsened. This mirrored a previous suicide attempt (in which the patient had attempted suicide by swallowing detergent after their delusions worsened). An entry in the diagnostic record made nine days before the event mentioned the worsening delusions, and an entry in the nursing record mentioned that the patient had bought detergent. However, due to turnover in ward staff over time, it may have been difficult for them to piece the disparate information together and identify the suicidal pattern.

In the first category (‘omission in diagnostic records during admission’), the following three sub-categories pertained to the same patient: [omission in patient information during readmission], [abridging report on suicide risk], and [factors influencing the ward staff’s judgements and behaviours]. The patient in question had been readmitted soon after being discharged, which may explain why the staff decided to cut corners and abridge the diagnostic records. Although the patient had evidently been readmitted because of severe suicidal ideation, there was just a brief, concise record stating that the patient had denied having such an ideation. On the second day after readmission, the ward received a report from the family referring to the patient’s suicidal ideation. However, the staff never discussed it with the patient. On the third day, the patient was absent from their room when the nurse came to deliver their medication. The nurse did not search for the patient right away; she first finished delivering medication to the other patients. Eventually, the nurse searched for the absconding patient and found them inside the ward lavatory. It was suicide by hanging. During Analysis 2, the professionals suggested that the omission of the diagnostic record may have prevented the hospital staff from appreciating the severity of the suicide risk, and that this complacency may explain why the nurse had delayed the initial response to the patient's absence.

One of the sub-categories of ‘communication lapse during transfer’ was [hard to relay complex information such as the principal doctor's decision-making process]. This sub-category pertained to a patient who had been transferred from a seclusion room in an open ward to the general area of the closed ward for the patient’s own protection following a suicide attempt. While bathing alone, the patient died by suicide by swallowing a substance. During Analysis 2, the professionals suggested that the staff at the closed ward, in whose care the patient had been transferred, may have mistakenly assumed that the patient no longer needed to be in seclusion because their symptoms had improved.

## Discussion

Assessing the suicide risk, which is non-constant and fluctuates over time, is essential in enabling timely intervention, and is therefore a top clinical priority. Of the cases we analysed, 61.1% of the patients died by suicide despite the absence of any verbalised references to suicidal ideation or exhibitions of suicidal behaviour in the 14 days leading up to the event. It is therefore crucial to look for signs of suicide other than explicit suicidal ideation statements or behaviour.

In this study, we retrospectively analysed the signs of imminent suicide risk that were present in psychiatric patients during the 14 days before their fatal suicide attempt, using diagnostic and nursing records pertaining to that period. In Table 2, we listed the items that describe signs. However, not all these items imply imminent suicide risk in themselves. Some of the items can be more accurately described as the lack of protective factors rather than imminent suicide risk. Three notable examples are the major category ‘signs from response to treatment’, the sub-category [symptoms] (in ‘signs emanating from the patient’), and the sub-category [attitude inferred from integrated information] (in ‘signs inferred from multiple sources of information’). These items imply that the person’s psychiatric condition has failed to improve sufficiently or has deteriorated, that the person is mentally unstable, or has a personality pathology. They do not directly imply imminent suicide risk to the extent that certain other items, such as <suicide attempt> or <accesses means of suicide>, do, and they are apt to occur in non-suicidal patients, too. Thus, to assess whether an item implies suicide risk, one must consider the specific details and severity of the case in question. Auditory hallucinations, for example, are more likely to imply suicide risk if they comprise suicide-related commands^16)^. All items under [nonverbal information], such as <evasiveness> and <poor treatment motivation>, describe, in our opinion, behaviours and attitudes that suggest the absence of protective factors. That is, inasmuch as the behaviours and attitudes that these items describe indicate a lack of readiness for treatment, they could be treated as evidence of the lack of the kind of protective factors discussed by Britton et al. (2020), which include a readiness to start finding value in one's life and to participate in one's own treatment^17)^.

The items that indicate the signs of imminent suicide risk in Table 2 represent the perspectives of the staff rather than those of the patients. The five major categories our analysis yielded reveal that suicide risk information can be obtained from ‘signs emanating from the patient’, ‘signs gleaned through engagement’, ‘signs from response to treatment’ (signs that occur as a result of medical intervention), ‘signs associated with reports from the family’, and ‘signs inferred from multiple sources of information’ (denoting the need to base decisions on multiple kinds of information). In psychiatric wards, not only the principal doctor and nurse in charge, but also staff from different professions are stationed to provide care collectively. The perspectives extracted in our analysis can be particularly useful to staff other than those who are immediately in charge of patients. To such staff, the perspectives provide observational insights on the patients and can serve as criteria in deciding on the kinds of information to include (and excluding) while reporting to medical teams.

In-patient suicide is an example of a sentinel event─a sudden and unanticipated event or complication in a healthcare setting that results in death or serious injury. We used the fishbone diagram, which is an example of a sequential accident model. Other models for analysing accidents extend their focus to epidemiological and systemic accident models^18)^. There is a drive for suicide in patient, so such issues with the handling of information by staff should be seen less as a factor that causes a suicide event and more as a factor pertaining to the defence barriers in an epidemiological accident model. Such models envisage effective communication among healthcare workers as a defence against accidents. Defences must be as solid as possible to prevent accidents. We extracted five issues pertaining to how hospital staff handle information: ‘omission in diagnostic records during admission’, ‘omission in conference records’, ‘communication lapse during transfer’, ‘need for integrated information’, and ‘systemic issues’. Information may be omitted during admission, conferences, or ward transfers because the staff are busy or have a defensive mental state, or the hospital is understaffed. To address these issues, it may be useful to build a system that makes omissions impossible, such as by standardising the reporting format. The staff may be particularly tempted to omit or abridge information in the case of readmission. They may do so out of complacency, believing that the facts on the readmitted patient are already well known. The fact that a patient is being readmitted because of the lack of improvement in their condition may create a sense of helplessness among the staff, which may, in turn, dull their alertness to the suicide risk. Thus, hospitals must impress upon the staff the importance of recording suicide risk information during readmission. We found that communication lapses occur during ward transfers because it is [hard to relay complex information such as the principal doctor’s decision-making process]. It is difficult to explicate the principal doctor’s often inscrutable thinking to the staff at the destination ward. For example, the doctor may give the staff seemingly contradictory instructions to transfer a patient from a seclusion room in an open ward to a general area in a closed ward, i.e. to increase or decrease the level of protection. It is therefore essential to develop strategies to convey this information. For example, when the staff in the destination ward prepares a nursing plan for the transferee, the principal doctor and staff from the original ward can participate in the process. The ‘need for integrated information’ implies the need for collating information from different staff members and evaluating multiple sources of information in an integrated manner. This task poses a major challenge in that it is subject to human factors such as how busy one is and what their career and abilities are. Possible strategies toward addressing ‘systemic issues’ include introducing an electronic medical record and displaying information on past suicidal behaviour patterns in places where the staff can easily see it.

This study has four limitations. First, the results have limited transferability given the small size and peculiarity of the sample. Second, our analysis was unable to cover factors that were not included in the records. Third, as the cases were retrospectively analysed by parties who were not involved, the analysis could not cover the perspectives of psycho-dynamics and group dynamics between each case and staff and the participant observation of the staff. Fourth, another aspect that was not covered in the analysis are factors that were related to the hospital setting during the period in question. Examples include the conditions of in-patients, staff numbers and transfers, and relationships among the staff and between the frontline and other staff in the hierarchy of the organisation. Addressing these limitations in future research can provide insights on the signs of imminent suicide risk among psychiatric in-patients.

## Funding

No funding was received.

## Author contributions

HS, IU, NS, and TF designed this study. HS, IU, CS, SS, and KY carried out the analyses. HS summarised the result and wrote the manuscript with the support and guidance from IU, NS, and TO. IU, NS, and TF supervised the project. All authors read and approved this manuscript.

## Conflicts of interest statement

The authors declare that there are no conflicts of interest.
